# New Vaccine Technologies to Combat Outbreak Situations

**DOI:** 10.3389/fimmu.2018.01963

**Published:** 2018-09-19

**Authors:** Susanne Rauch, Edith Jasny, Kim E. Schmidt, Benjamin Petsch

**Affiliations:** CureVac AG, Tuebingen, Germany

**Keywords:** viral vector vaccine, DNA vaccine, mRNA vaccine, pandemics, vaccine development

## Abstract

Ever since the development of the first vaccine more than 200 years ago, vaccinations have greatly decreased the burden of infectious diseases worldwide, famously leading to the eradication of small pox and allowing the restriction of diseases such as polio, tetanus, diphtheria, and measles. A multitude of research efforts focuses on the improvement of established and the discovery of new vaccines such as the HPV (human papilloma virus) vaccine in 2006. However, radical changes in the density, age distribution and traveling habits of the population worldwide as well as the changing climate favor the emergence of old and new pathogens that bear the risk of becoming pandemic threats. In recent years, the rapid spread of severe infections such as HIV, SARS, Ebola, and Zika have highlighted the dire need for global preparedness for pandemics, which necessitates the extremely rapid development and comprehensive distribution of vaccines against potentially previously unknown pathogens. What is more, the emergence of antibiotic resistant bacteria calls for new approaches to prevent infections. Given these changes, established methods for the identification of new vaccine candidates are no longer sufficient to ensure global protection. Hence, new vaccine technologies able to achieve rapid development as well as large scale production are of pivotal importance. This review will discuss viral vector and nucleic acid-based vaccines (DNA and mRNA vaccines) as new approaches that might be able to tackle these challenges to global health.

## Introduction

The world population has grown to 7.6 billion people in 2018, more than half of which live in densely populated urban settings. Travel habits have changed radically; the number of people traveling by plane is growing each year and amounted to a total of 3.7 billion in 2016^1^. The high population density, as well as the extreme increase of contact between people from virtually all areas of the world highly favor global spreading of pathogens. This pandemic risk is further increased by the climate change that influences the distribution, abundance, and prevalence of pathogen-bearing vectors, promoting infections with a range of vector-borne diseases. The occurrence of pandemic outbreaks in the past decades has clearly demonstrated the reality of global pandemic threats.

Human immunodeficiency virus (**HIV**), the causative agent of acquired immunodeficiency syndrome (AIDS), represents a zoonosis from non-human primates in West-central Africa and has claimed more than 35 million lives since its discovery in 1983^2^. Despite the development of effective highly active anti-retroviral therapy (HAART), drugs are cost intensive and access to therapy remains problematic in resource limited settings in which the majority of infections occur. Development of a direly needed vaccine against HIV has proven extremely difficult and identification of a suitable method for generating such a vaccine remains the focus of research.

**Influenza A viruses** occur in annual seasonal outbreaks. However, their ability to infect a variety of different species as well as their high genomic variability additionally bears the constant risk of a zoonosis introducing a virus with completely new immunogenic properties into the human population. While the occurrence of a future influenza pandemic is almost certain, it is impossible to predict the characteristics of the virus and the severity of the symptoms it induces. This unpredictability can be illustrated by the “swine flu” (H1N1pdm09) on the one hand, that led to a phase 6 pandemic alert declared by the WHO in 2009 but caused relatively mild symptoms and the 1918 influenza A H1N1 pandemic (“Spanish flu”) on the other hand, that resulted in the deaths of around 50 million people (1). Currently licensed seasonal influenza vaccines are specific for pre-defined viral strains and are unable to protect against a future pandemic. Hence, new vaccine technologies able to induce broad protection against influenza A viruses are urgently required.

**Severe acute respiratory syndrome (SARS)** first occurred in China in 2002 and was caused by a novel coronavirus (CoV) that likely originated in bats (2, 3). SARS CoV caused a global outbreak with 8,000 infected patients, leading to 774 deaths in 26 countries (4). A notable aspect of the SARS epidemic was the efficacy of containment measures that halted the spread of disease. Following this, ongoing efforts to develop a vaccine against SARV-CoV were discontinued (5). In 2012, a new coronavirus appeared in Saudi Arabia causing **Middle East respiratory syndrome (MERS)**. Like SARS CoV, the virus originated in bats and likely spread to humans via infected dromedary camels. According to the WHO, there have been 2,143 confirmed cases of MERS, with 750 deaths in 27 countries since 2012.^3^ A variety of research activities are currently ongoing to develop a vaccine against MERS CoV. However, a licensed vaccine is not yet available.

**Ebolaviruses** belong to the family *Filoviridae* (consisting of the two genera Ebolavirus and Marburgvirus) that cause hemorrhagic fever with a high mortality rate and whose natural reservoir is believed to be in bats (6). Since the first documented Ebolavirus outbreaks in 1976, Ebolaviruses have emerged periodically in outbreaks that mostly occurred in Central African countries.^4^ During this period, attempts to develop a vaccine against Ebolaviruses were made but remained at research and early development stages. However, when Ebola virus appeared in West Africa in late 2013, it hit a region heavily affected by poverty and armed conflicts, in which many factors, among them a dysfunctional health system, contributed to the inability to control the virus. The 2013–2016 Ebola crisis represented the first epidemic caused by an Ebolavirus with 28,616 cases and 11,310 deaths reported.^5^ At late stages of the epidemic, several vaccine candidates were tested in clinical trials, the most advanced of which (rVSV-ZEBOV) showed clinical efficacy in a ring-vaccination clinical trial (7).

The vector borne diseases **Dengue**, **Chikungunya**, and **Zika** are transmitted by species of *Aedes* mosquitoes and induce similar symptoms such as fever and severe joint pain. At present, more than half of the world's population lives in areas where these mosquito species are present. Infection rates for all these viruses have increased dramatically in the last decades: according to the WHO, cases of dengue fever have risen 30-fold in the past 50 years. Zika virus was first identified in non-human primates in Uganda in 1947 (8) and has since caused several outbreaks in different areas with reported mild symptoms such as self-limiting febrile illness. Since 2014, however, outbreaks in Asia and the Americas have been linked to severe clinical manifestations, including Guillain–Barré syndrome in adults and congenital abnormalities, including microcephaly, following infection during pregnancy. A possible explanation for the emergence of these aggravated symptoms could be mutations introduced in the virus that allowed adaptation to the new environment and resulted in changes to pathogenicity. The occurrence of around one million laboratory confirmed cases of Zika in South America, with over 4,000 cases of microcephaly led to the declaration of a Public Health Emergency of International Concern (PHEIC) in February 2016 (9). The Zika crisis has prompted the accelerated development of vaccines against Zika virus, seven of which have entered clinical trials (10). Likewise, several clinical trials are currently ongoing testing different technologies for a vaccine against Chikungunya or Dengue. However, with the exception of a vaccine against Dengue (Dengvaxia® developed by Sanofi Pasteur) no other vaccine has been licensed for these diseases. Of note, Dengvaxia® has recently been associated with increased risk of more severe disease in subjects who had never been exposed to the virus (11). In April 2018, the WHO recommended a pre-vaccination screening strategy, in which Dengvaxia® is only used in dengue-seropositive individuals.^6^

In addition to pandemic threats, the list of **multi drug resistant (MDR) organisms** is ever-growing, favored by the misuse and overuse of antibiotics. This holds true for the use of antibiotics in both humans and, even more problematically, in animals, where antibiotics are routinely used for prevention of disease and promotion of growth in livestock. MDR organisms, such as methicillin-resistant *Staphylococcus aureus* (MRSA) or multidrug-resistant tuberculosis (MDR-TB) are becoming a serious threat to global public health. According to WHO estimates, 490,000 new cases of MDR-TB were registered in 2016, of which only 54% could be successfully treated. Again, the solution to this growing threat could be the development of efficient vaccines to prevent MDR organisms from further spread.

## The challenges of vaccine development in outbreak situations

Conventional vaccines, developed by attenuating or inactivating the respective pathogen, have successfully decreased the burden of a number of infectious diseases in the past, leading to the eradication of small pox and significantly restricting diseases such as polio, tetanus, diphtheria, and measles. However, established methods may not always be suitable or even feasible in outbreak situations. Live attenuated vaccines generally bear the risk of reversion, rendering this approach unfavorable for highly pathogenic, possibly largely uncharacterized organisms. Inactivation may not induce protective responses, as is the case for Ebola (12) or can even lead to undesired effects, like formalin-inactivated RSV (respiratory syncytial virus) that induced exacerbated disease upon wildtype RSV infection in clinical trials in the 1960s (13). Furthermore, outbreak scenarios may limit conventional vaccine development in terms of producibility. Since these methods require whole pathogen cultivation and propagation, vaccine production may be hampered by factors such as difficult or impossible cultivation of the respective pathogen under *in vitro* conditions or the requirement of a high biosafety level and specialized labs for cultivation. Hence, new and highly versatile approaches that are independent of whole pathogen cultivation are required to effectively and quickly combat outbreak situations.

In order to proof effective against an upcoming pandemic, these new technologies need to overcome a number of challenges. The unpredictable nature of emerging pathogens represents one of the pivotal problems for pandemic preparedness. Zoonoses present a constant threat to introduce a previously uncharacterized pathogen into the population, as was the case for HIV as well as for SARS and MERS CoV. The outbreaks caused by pandemic influenza virus demonstrate the potential of a known pathogen to mutate and adapt to a new host or environment, with unpredictable outcomes for its immunogenic properties and the severity of symptoms it induces. As demonstrated by the recent epidemics and pandemics, the risk of such events is highest for RNA viruses, whose high mutation rates favor adaptability.

Since the vaccine targets remain undefined before an outbreak occurs, time remains one of the major hurdles for effective vaccine development. Currently, the average development time for conventional vaccines from preclinical phase is more than 10 years (14), highlighting the dire need for new approaches that allow extremely fast development and licensing to prevent an emerging outbreak from global spread.

A further major problem is the cost associated with vaccine development and production: using established technologies, development of a new vaccine candidate is estimated to amount to more than 500 million USD, with further expenses to establish facilities and equipment ranging from 50 to 700 million USD (15). While some costs for vaccine development cannot be avoided in order to keep the required safety standards, the need for dedicated production processes and facilities for each vaccine in most conventional vaccine technologies keeps validation and production costs high. Especially considering resource limited settings such as the 2013–2016 Ebola crisis and the fact that outbreak situations represent niche markets, new technologies are required to support more cost effective vaccine production.

A further issue is production capacities of established methods, which are often insufficient to support global vaccination. Even if the potential threat is known and vaccine manufacturing technologies are established, like for pandemic influenza vaccine, production capacity to meet peak demands during a pandemic remains problematic. Thanks to efforts coordinated by the WHO, the potential production capacity for pandemic influenza vaccines in 2015 could in theory support the vaccination of 43% of the population with two doses of vaccine (16). However, the global distribution of vaccine production is far from equal between industrial nations and the developing world: according to a survey made in 2015, only 5% of influenza vaccine doses were distributed among Southeast Asia, Eastern Mediterranean, and Africa WHO regions, which comprise about half of the world's population (17). In addition, most currently licensed vaccines would take 5–3 months between identification of a pandemic influenza and vaccine distribution, which would give a pandemic virus ample time for global spread. Hence, technologies that enable fast production of large amounts of vaccine are direly needed in the face of pandemic threats.

Efforts to meet these challenges are made by monitoring viruses with high pandemic potential and programs, most notably Coalition for Epidemic Preparedness Innovations (CEPI), that finances and develops vaccines against likely pandemic threats.

## Vaccine technologies

**Figure 1:**
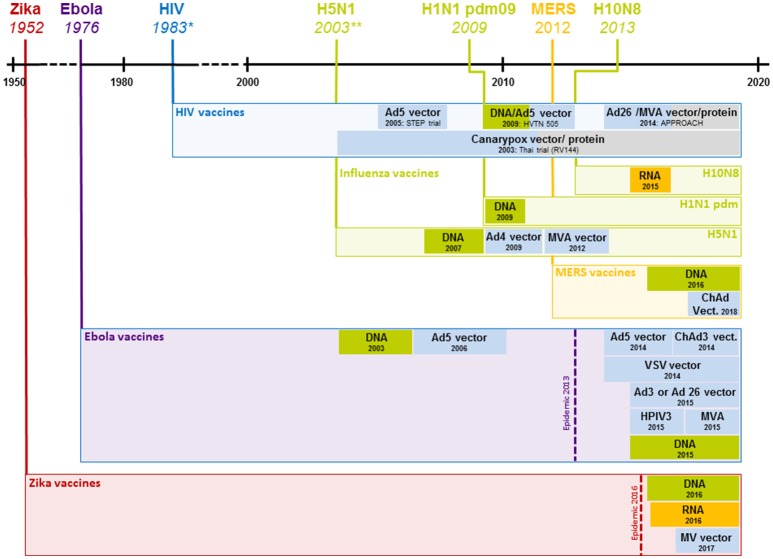
Clinical development of vaccines against recent outbreaks. The timeline above indicates the year a given virus started spreading in the human population; boxes below represent the start of clinical vaccine development and the employed technology (shown exclusively for viral vector and nucleic acid based vaccines). For HIV, only select studies that represent major advances are shown. ^*^1983 represents the year the HI virus was discovered; the virus likely started spreading at the beginning of the twentieth century. ^**^2003 represents the year H5N1 caused rising numbers of infections, the first H5N1 infection in a human was registered in 1997. Ad4, 5, 26, human adenovirus type 4, 5 or 26; ChAd, chimpanzee adenovirus; HIV, human immunodeficiency virus; H5N1, influenza H5N1; H1N1 pdm09, influenza H1N1 2009 “swine flu”; H10N8, influenza H10N8; DNA, deoxyribonucleic acid based vaccine, MVA, modified vaccinia Ankara; RNA, ribonucleic acid based vaccine; VSV, vesicular stomatitis virus; HPIV3, human parainfluenza virus type 3; MV, measles virus.

The past decades have witnessed the development of a wide array of new vaccination technologies ranging from targeted attenuation techniques of live pathogens to the delivery of biologically engineered protein and peptide antigens as well as viral vector and nucleic acid based antigens. Many of these technologies have yielded highly promising results which are discussed in excellent reviews elsewhere (18–21). Here, we will focus on the discussion of viral vector and nucleic acid based vaccines that have shown promise for offering solutions to the challenges of vaccine development. In order to visualize the time required between the occurrence of recent outbreaks and the onset of clinical trials, Figure 1 depicts an overview of the most important pandemics in relation to the start of clinical trials using different viral vector and nucleic acid based vaccines.

### Viral vector based vaccines

Viral vector based vaccines, that rely on the delivery of one or more antigens encoded in the context of an unrelated, modified virus, represent a highly versatile platform that offers many advantages over more established vaccine technologies. This technology either employs live (replicating but often attenuated) or non-replicating vectors. Research conducted since the 1980s has established a variety of viruses as vaccine vectors by engineering them to encode for heterologous antigens that are shuttled into the host cells by the vector. Upon delivery, antigens are expressed and the host is able to induce immune responses against the respective target pathogen (22).

#### Description and mode of action

A wide array of different viruses has been employed as a basis for constructing viral vector based vaccines (23). Among others, these vectors include adenoviruses, parvoviruses (e.g., adeno-associated viruses, AAV), togaviruses (e.g., Semliki Forest virus), paramyxoviruses (e.g., measles virus, Newcastle disease virus or human parainfluenza virus), rhabdoviruses (e.g., vesicular stomatitis virus, VSV), and poxviruses (e.g., Modified vaccinia Ankara, MVA). Since a comprehensive discussion of all these vectors would exceed the scope of this review, we will only describe some commonly used viral vectors, i.e., adenovirus, measles virus, and VSV in some detail, whose use in clinical studies will be discussed below.

**Adenovirus (Ad) vectors** are among the most commonly employed viral vectors, with vast amounts of both preclinical and clinical studies assessing their protective efficacy against a variety of infectious diseases available. *Adenoviridae* are non-enveloped viruses with an icosahedral capsid and a linear double-stranded DNA genome, whose size ranges from 30 to 40 kb. Next to a multitude of adenoviruses occurring in different animal species, there are 57 identified human adenovirus that are classified into seven species A–G. Adenoviral receptors are expressed on the surface of most human cells, allowing a broad tissue tropism of the virus (24).

Ad based vaccines can be constructed as replication-competent or replication-defective vectors, which are generated by replacing the E1A and E1B (early transcript 1 A and B) genomic region by an antigen expression cassette, thereby abolishing the viral ability to replicate (25). In addition, the viral E3 and E4 genes are frequently deleted to prevent elimination of Ad infected cells by the immune system and leaky expression of the inserted antigen, respectively (25). Since adenoviruses shuttle their genome in the nucleus of the host cell for transcription and replication, the risk of genomic integration exists, however, the vector predominantly remains episomal (24). Adenoviral vectors are able to stably express inserts of up to 8 kb, supporting the expression of most target antigens as well as multivalent or multi-pathogen vaccines (26). The vector is easily manipulated by insertion of a transgene cassette into the viral backbone via homologous recombination or through a direct cloning step *in vitro* (27). Adenoviral vectors can be manufactured in mammalian cell culture systems, most commonly using HEK 293 cells that provide E1 protein in trans to allow viral replication. These production systems support high viral yields at relatively low production costs, but amplification of viral seed requires biosafety level 2 (BSL2) facilities (23).

Adenoviral vectors are able to induce potent antibody as well as T cell responses with variations in the immune response depending on the serotype employed (28). Replication-deficient Ad5, one of the most widely used adenoviral vectors, is able to induce exceptionally potent CD8^+^ T cell as well as antibody responses (29). However, the widespread pre-existing immunity to this virus in the human population, that can inhibit transgene expression and inactivate the viral vector, hampers its clinical use (30). This issue has been met by developing adenoviral vectors of non-human origin, such as the chimpanzee virus derived vector ChAd63 (31). An alternative approach is the selection of rare serotypes with low prevalence in humans such as Ad26 or Ad35 (32) which induce enhanced memory and more poly-functional CD8^+^ T cells compared to Ad5 (28).

**Measles virus** (MV), a common human pathogen, belongs to the family of *Paramyxoviridae*. MV is an enveloped virus with a non-segmented, negative-sense, single-stranded RNA genome of ~16 kb. Measles virus vaccines have been generated by serial passaging of infectious virus through different cell lines resulting in a live attenuated virus that is replication deficient in humans. The introduction of numerous mutations in this process has established a highly stable vaccine for which reversion to pathogenicity has never been observed (33). Moreover, MV is unable to integrate into the host genome and a lyophilization process for MV vaccine has been established, increasing the thermostability of the naturally unstable virus. MV vaccine induces extremely durable responses with both antibodies and CD8^+^ cell persisting as long as 25 years post vaccination (34).

Due to the helical nature of the ribonucleoprotein (RNP) complex, the viral genome is highly flexible and accepts insertions of up to 6 kb, as long as the total number of nucleotides in the genome can be divided by 6 (“rule of six”). The ability to accept relatively large transgenes offers the opportunity to generate multi-pathogen or multivalent vaccines (26). However, the need to rescue the negative-sense RNA genome by reverse genetics renders manufacturing of the virus and the insertion of the transgene more complex compared to other viral vectors. Several ways to generate transgene expressing MV have been described and transgene cassettes can be inserted at different positions in the viral genome (35). MV vaccines can be grown in chick embryonic fibroblasts or cell lines such as Vero or MRC-5 cells and manufacturing processes for clinical use are well-established. However, the manufacturing and bulk vaccine production requires BSL2 facilities, which might restrict availability of manufacturing facilities in an outbreak setting.

Recombinant measles viruses are able to induce high levels of both humoral and cellular immune responses against the transgene (33). Importantly, MV is able to infect cells of the immune system, including macrophages and dendritic cells, thus supporting delivery of target antigens directly to antigen-presenting cells (36). T cell-mediated responses to MV are dominated by a CD4^+^ phenotype, unlike the more CD8^+^ dominated response to adenoviral vectors, which might be a consideration for vaccine development. Since live attenuated MV is routinely used as a vaccine in child immunization programs in many countries, pre-existing immunity to MV as a viral vector has been raised as a concern. However, studies in mice and macaques showed no impact of previous MV exposure on transgene immunity (29). In agreement with animal studies, a clinical study conducted in the context of a MV vaccine against CHIKV likewise demonstrated that anti-vector immunity did not compromise vaccine efficacy (37).

**Vesicular Stomatitis Virus (VSV)**, a member of the *Rhabdoviridae* family, is an enveloped virus containing a single stranded, negative-sense RNA of ~11 kb. The virus naturally infects livestock with sporadic infections found in humans (38). The resulting low risk of pre-existing immunity and the lack of a DNA intermediate during viral replication makes VSV attractive as a safe vaccine for applications in humans. The establishment of a reverse genetic system for VSV in 1995 has allowed manipulation and propagation of the virus (39). VSV is generally employed as an attenuated vector, which is achieved by different methods, such as introducing mutations in the viral matrix (M) protein, rearranging the order of the viral protein, insertion of non-viral proteins and partial or complete deletion of the viral glycoprotein (G), the determinant for viral infectivity (40). Attenuation is essential for vaccine safety, since neurovirulence of the wild-type VSV has been detected upon intracranial inoculation in animal models (41). Transgenes can be inserted at different positions in the viral genome resulting in varying levels of transgene expression. A common method for transgene insertion replaces the G protein, which alters tissue tropism of the virus (42). The amount of additional genomic material stably accepted in the genome is 4–5 kb (29). VSV can be grown to high titers in most mammalian and insect cell lines. Depending on the way the virus has been manipulated, methods for viral propagation may vary.

VSV induces robust antigen-specific neutralizing antibody responses. Modest CD8^+^ and CD4^+^ T cell immunity has been described in several studies, however, the asset of the vaccine is the effective induction of humoral responses (29).

#### Delivery of viral vector based vaccines

Administration of viral-vectors can take place by different routes: next to intramuscular vaccination, intranasal (43), intradermal (44, 45), and oral vaccination (46) have been tested for different viruses in clinical studies. Next to the ability of the employed virus to infect certain tissues, the choice of immunization route is dependent on several considerations. The route of administration affects the quality of the induced immune response and the choice of application route thus depends on the target pathogen, i.e., if a mucosal response is required for inducing protection, oral or nasal delivery of the vaccine may be preferable over parenteral applications. In addition, the route of administration needs to be reliable and easy to perform in an outbreak situation, arguing for established routes of vaccination such as oral or intramuscular administration (47).

Since viral-vectors are complex vaccines that induce strong immune responses, the use of additional adjuvants is generally not required. Some clinical studies have tested recombinant viral vaccines in combination with additional immune-stimulating components (48, 49) but found no increase in immunity in the adjuvanted group (49). Nevertheless, the modification of the immunological compartment introduced by an adjuvant might still prove beneficial in the context of some viral vectors.

#### Advantages and disadvantages

Given the large amount of different viral vectors available and the vast knowledge gathered about their manipulation and function as immunogens, viral vector based vaccines represent a valuable and highly versatile platform for vaccine development. Viral genomes can be manipulated to express any antigen of choice and the ability to stably accept relatively large insertions in their genome supports the development of a large variety of vaccines. Delivery of the target antigen as genetic information allows faithful antigen generation, targeting and processing, i.e., correct protein folding, multimerization, modifications such as glycosylation, and specific targeting in the cell are ensured. Of note, this mostly holds true for viral target antigens derived from human pathogens which are expressed in their natural environment, whereas isolated bacterial or parasitic antigens might be localized and processed differently in mammalian cells compared to their natural host. Viral vectors induce stimuli in the target cells that mimic natural infection, thereby inducing potent immune responses. Hence, viral vector based vaccines can be delivered without additional adjuvants and, with variations depending on which vector is employed (see above), strong antigen-specific cellular and humoral immune responses against the target antigen can be induced. Strategies to achieve replication incompetency or attenuation of modern viral vectors generally ensure a good safety profile of viral vector based vaccines. For most commonly employed viral vector based vaccines, high yield production processes with means of upscaling have been established, supporting the use of these technologies for pandemic settings.

Despite many advantages, several aspects have to be considered when developing a viral vector based vaccine. Firstly, viral vectors are genetically modified organisms (GMOs) and are therefore considered a potential risks to human health and environment associated with the release of these organisms. European regulatory agencies require environmental risks assessment (ERA) to evaluate potential environmental and health risks posed by the GMO (50). In the USA, the FDA has published guidelines for Environmental Assessments (EA).^7^ What is more, the use of viral vector based vaccines raises safety concerns for use in humans, such as potential integration into the host genome or too high or persistent replication of attenuated vaccines, that need to be carefully assessed before entry into, as well as during clinical development. These concerns are not only important in terms of safety, but might also lead to delays of clinical studies in case of a pandemic.

In terms of vaccine manufacturing, each viral system requires different cellular systems for high yield propagation, necessitating different manufacturing facilities for each viral vector platform. As viruses may undergo recombination during production, great care must be taken to keep cell cultures free of material that can lead to the emergence of recombined and uncharacterized pathogens (51). In general, the presence of adventitious agents, i.e., microorganisms that may have been unintentionally introduced into the manufacturing process, needs to be assessed vigorously during vaccine manufacturing (52). Since production of viral vector based vaccines is a complex process that often requires a multitude of components of human or animal origin, such as cell substrates, porcine trypsin or bovine serum, the need to exclude contaminants requires extensive testing during various steps of the manufacturing process. Indeed, several examples for contaminants in viral vaccines, such as porcine circovirus contaminations in rotavirus vaccines, have highlighted the reality of this risk (53). These factors make production of viral vector based vaccines a highly complex and comparatively cost-intensive process. If the viral vector is derived from a virus able to infect humans, the effect of pre-existing immunity on vector immunogenicity has to be addressed. Depending on the vector, this effect may or may not hamper immune responses, as was the case for Ad5 and MV vectors, respectively (see above). Dampening of immune responses by pre-existing immunity may necessitate time and cost intensive screening procedures before clinical trials and compromise the use of a given vector for further indications in the same vaccinee.

#### Viral vector based vaccines in potential pandemic settings using ebola virus as an example

Viral vector based vaccines have been employed for the development of vaccines against many different pathogens in a vast number of preclinical and clinical studies. However, so far only one viral vector based vaccine, i.e., Dengvaxia, which is a recombinant Dengue vaccine based on the yellow fever attenuated strain 17D, has been licensed for human use. More comprehensive summaries of their applications in the context of prophylactic vaccines are published elsewhere (23, 29). In this review, we will focus on two exemplary vector based vaccines developed in the context of the recent Ebola pandemic in order to highlight some of the advantages and disadvantages of this technology for outbreak situations.

First studies employing viral vector based approaches to develop vaccines against **Ebolaviruses** started as early as the 1990s. However, most approaches were still in preclinical stages when the Ebola pandemic emerged in 2014. Viral vector based vaccines against Ebolaviruses have been tested in the context of non-replicative vectors such as modified vaccinia strain Ankara (MVA), human adenovirus (Ad) and replication-defective recombinant chimpanzee adenovirus type3 (ChAd3 vaccine) as well as replication competent vectors including VSV-EBOV, human parainfluenza virus type 3 (HPIV3), recombinant cytomegalovirus (rCMV), and recombinant rabies virus (RABV). Clinical trials were conducted for VSV-EBOV, ChAd3 vaccine, Ad26-EBOV, Ad5-EBOV, HPIV3, and MVA-vector vaccine (54). These vaccines rely on vector based expression of the viral glycprotein (GP), the only surface protein and single target of neutralizing antibodies alone or in combination with additional viral proteins. Here, we will focus on the discussion of two adenoviruses, i.e., Ad5 and ChAd3, and VSV-EBOV vectors as three of the earliest vector based vaccines to enter clinical trials upon the 2014 pandemic.

The first adenovirus based vaccine against Ebola, replication defective Ad5 expressing EBOV GP, was described in 2000 and tested in combination with DNA vector vaccination in non-human primates (NHPs). Vaccination was found to be protective but required long vaccination schedules (55). This vaccine was further developed by generating a vector expressing both GP and the nucleoprotein (NP) to enhance T cell responses. Indeed, vaccination with this vector resulted in complete protection in NHPs upon a single vaccination. Protection was found to correlate with both the generation of specific CD8^+^ T cell and antibody responses (56). Further studies employed an Ad5 vector developed by Crucell Holland BV that expressed GPs from two Ebolavirus subspecies [Ebola virus (EBOV) and Sudan Ebolavirus (SUDV)] featuring a point mutation that reduced protein cytotoxicity. The vaccine was found to be protective in NHPs while allowing deletion of NP from the construct as well as dose sparing (57). Given these encouraging results, a clinical trial (NCT00374309) was initiated in 2006 (Table 1). This study showed safety as well as the induction of antibody and T cell responses, but no significant generation of virus neutralizing titers (58). Importantly, this study also demonstrated that the induction of antibodies was reduced in participants with pre-existing immunity against Ad5. Given the high prevalence of 60–90% of Ad5 in the human population, this finding might compromise the use of Ad5 for the development of human vaccines. Upon the outbreak of the Ebola pandemic in 2014, a new Ad5 based vaccine was developed in a joint effort by the Beijing Institute of Biotechnology and Tianjin CanSino Biotechnology Inc. This vaccine was the first to incorporate the GP of the 2014 epidemic Ebola strain and was produced as a lyophilized powder that facilitated vaccine transport and storage by allowing storage at 2–8°C. A phase I clinical trial initiated at the end of 2014 (NCT02326194), showed no serious adverse events, although higher incidences of injection-site reactions were associated with higher Ad5 doses (Table 1). Importantly, this study showed that high doses of Ad5 vector were able to overcome the negative effects of pre-existing immunity, as participants with a high baseline concentration of Ad5 neutralizing antibodies still induced robust GP-specific antibody and T cell responses (59). A phase II clinical study (NCT02575456) testing the Ad5 viral vector was initiated in Sierra Leone in October 2015 (Table 1), results are not yet publicly available.

**Table 1:** Exemplary clinical trials employing viral vector based vaccines in the context of Ebola vaccine development.

**Study start**	***N***	**Vaccine and delivery**	**Outcome**
**NCT00374309**			**Phase I**
Sept 2006	31	**Ad5** IM 2 × 10^9^ or 2 × 10^10^ VP Antigen: GP EBOV and SUDV	**Safety:** Acceptable safety profile **Immunogenicity:** - Antibody responses in 100% (SUDV GP) and 55% (EBOV GP) of subjects in the higher dose group- No significant induction of VNTs - T cell responses in 82% (SUDV GP) and 64% (EBOV GP) **Of note:** Reduced immunogenicity in participants with pre-existing immunity against Ad5
**NCT02326194**			**Phase I**
Dec 2014	120	**Ad5** IM 4 × 10^10^ or 1.6 × 10^11^ VP Antigen: GP EBOV (2014)	**Safety**: No serious adverse events. **Immunogenicity:** - Antibody responses in all but two participants (lower dose) and all (higher dose group) by d28 - Specific T cell responses (by ELISPOT and ICS); **Of note:** high dose of Ad5 vector able to overcome negative effects of pre-existing immunity
**NCT02575456**			**Phase II**
Oct 2015	500	**Ad5** IM 8 × 10^10^ or 1.6 × 10^11^ VP Antigen: GP EBOV (2014)	Results not yet publicly available
**NCT02269423; NCT02280408**			**Phase I**
Oct 2014	78	**VSV, attenuated** one or two doses IM 1 × 10^6^, 2 × 10^7^ and 1 × 10^8^ PFU Antigen: GP EBOV (1995)	**Safety:** Mild adverse events, no cases of arthritis **Immunogenicity:** - Antibody titers in all participants by day 28 - Increased levels of total and VNTs upon delivery of higher doses
NCT02231866; NCT02240875^*^; NCT02267109^*^			**Phase I**
Aug 2014–Aug 2017	325	**ChAd3, replication deficient** Single dose IM 1 × 10^10^, 2.0 × 10^10^, 2.5 × 10^10^, 5 × 10^10^, 1 × 10^11^, 2.0 × 10^11^ VP Antigen: GP EBOV (1976) ± GP SUDV (1977)	**Safety:** Acceptable safety profile, mild to moderate adverse events. **Immunogenicity:** - Antibody responses in almost all subjects; indications for durability (significant antibody titers detectable up to 48 weeks post vaccination) - VNTs in some subjects - Antigen-specific CD4^+^ and CD8^+^ T cells in some subjects - Increased immune responses upon MVA boost
NCT02289027; NCT02344407^**^; (NCT02485301); (NCT02548078)			**Phase I/II**
Oct 2014 - Nov 2015	5244	**ChAd3, replication deficient** Single dose IM 2.5 × 10^10^, 5 × 10^10^, 1 × 10^11^ VP Antigen: GP EBOV (1976)	**Safety:** NCT02289027: Acceptable safety profile NCT02344407: serious adverse events within 12 months after inj. in 8.0% (40/500) of participants (9.4% in rVSV-ZEBOV) **Immunogenicity:** NCT02289027 - Antibody responses peaked at d28 (51 μg/ml high dose group); still significantly over placebo at d180 (25.5 μg/ml) - CD4^+^ and CD8^+^ T cell responses in 57% (28/49) and 67% NCT02344407 - Antibody responses in 70.8 and 63.5% of the participants at 1 and 12 months, respectively (83.7 and 79.5% for VSV-ZEBOV)
**NCT02283099; NCT02296983; NCT02287480**			**Phase I; Phase I/II**
Nov 2014	158	**VSV, attenuated** single dose IM 3 × 10^5^, 3 × 10^6^, 1 × 10^7^, 2 × 10^7^, 5 × 10^7^ PFU Antigen: GP EBOV (1995)	**Safety:** Doses of 1 × 10^7^ PFU or higher: - Arthralgia in 22% (11/51) participants of Geneva cohort; arthritis confirmed in 9/ 11 cases; maculopapular rash in 27% (3/11) of these cases - Self-limiting cases of arthritis in 3.4% (2/60) participants in Germany and Kenya cohort Dose of 3 × 10^5^ PFU: - Reduced adverse events in mild to moderate range with arthralgia in 23% (13/56) participants **Immunogenicity:** - Antibody responses in all subjects; persisted for 6 months - Dose dep. VNTs in 85% (107/126) of vaccinees
**NCT02378753**			**Phase II/III**
March 2015	7651	**VSV, attenuated** single dose IM 2 × 10^7^ PFU Antigen: GP EBOV (1995)	**Safety:** Acceptable, one serious adverse event **Immunogenicity:** - Ring vaccination approach; 48 clusters (4,123 people) and 42 clusters (3528 people) randomly assigned to immediate and delayed vaccination (21 days later) - No cases of Ebola virus disease with symptom onset at least 10 days after randomization (immediate vaccination), 16 cases from seven clusters (delayed vaccination) 100% vaccination efficacy

This table exclusively lists exemplary clinical trials discussed in the text. Ad5, Adenovirus 5; EBOV, Ebola virus; GP, Glycoprotein; ICS, intracellular staining; IM, intramuscular; N, number of study participants; PFU, Plaque Forming Unit; SUDV, Sudan virus; VNT, virus neutralization titers; VSV, vesicular stomatitis virus; VP, viral particles.

* Boost with MVA based vaccine evaluated;

** *Direct comparison with rVSV-ZEBOV arm*.

In addition to Ad5 vector based strategies, limitations associated with the high prevalence of this virus in the human population are met in parallel approaches employing the far less prevalent Ad26 and Ad35 or related viruses such as chimpanzee derived adenoviruses (ChAd3). Especially ChAd3 is among the most widely evaluated vectors for the development of a vaccine against Ebola. Two vaccines developed by the NIAID VRC, i.e., replication defective ChAd3 encoding for EBOV GP alone or in combination with SUDV GP, were tested in preclinical studies which demonstrated complete protection in NHPs for both vaccines 5 weeks after single injection, using 10^10^ viral particles. However, immune responses waned several months after prime vaccination which could be prevented by boosting with MVA encoding for GPs from EBOV and SUDV (60). Starting in September 2014, both vaccines were tested in phase I clinical trials (NCT02231866, NCT02240875, and NCT02267109) demonstrating an acceptable safety profile of ChAd3 vectors, the induction of GP specific antibody responses in almost all subjects as well as T cell responses in a subset of study participants (61–63) (Table 1). ChAd3 encoding for EBOV GP has been moved on to phase II clinical studies and is licensed by GSK (64). Published results of a phase I/II clinical trial (NCT02289027) report immunogenicity in almost all vaccine recipients and significantly increased antibody responses in the vaccine group compared to the placebo group at 6 months (65) (Table 1). Importantly, the PREVAIL study (NCT02344407), a phase II clinical trial that directly compared ChAd3 and rVSV-ZEBOV based vaccines, demonstrated that both vaccines elicited immune responses one month after vaccination that were largely maintained through 12 months (66). In addition, further trials are evaluating a prime-boost regimen of ChAd3 followed by MVA vaccines (64). Overall, ChAd3 based vaccine appears to be a safe and efficacious candidate for Ebola vaccine development.

rVSV-ZEBOV currently represents the most promising candidate for the development of an effective vaccine against Ebolaviruses. This vaccine consists of a live attenuated VSV in which the VSV glycoprotein is removed and replaced with the GP from a 1995 EBOV strain. rVSV-ZEBOV was developed by the Canadian National Microbiology Laboratory and is now licensed to Merck. Preclinical studies published in 2004 and 2005, respectively, demonstrated complete protection from a lethal EBOV challenge infection in mice using a mouse-adapted strain (67) and NHPs with a single injection (68). rVSV-ZEBOV was demonstrated to be fully protective in NPHs when the vaccine was applied only seven days before challenge (69) and showed promise as a post-exposure prophylaxis in NHPs: injection with one or two doses of vaccine 1 or 24 h after EBOV exposure resulted in 33–67% protection (70). The vaccine was tested in ten completed phase I clinical trials with the earliest study having been initiated in October 2014 (71). First results from clinical studies (NCT02283099, NCT02287480, and NCT02296983) published in 2016 (72) showed robust and persistent induction of GP specific antibody responses as well as virus neutralizing titers with higher titers elicited in higher dose groups (Table 1). However, these studies also raised safety concerns: doses of 1 × 10^7^ PFU or higher were associated with the development arthritis lasting a median of 8 days. In addition, some participants experiencing arthralgia developed a maculopapular rash indicative of VSV replication and dissemination. Following this, the study was suspended and resumed one month later using a lower dose of 3 × 10^5^ PFU (NCT02287480). Reduction of viral titers employed for vaccination yielded reduced adverse events. However, while the frequency of GP specific antibody induction remained similar to cohorts vaccinated with higher doses (94%), levels of antibody responses were reduced.

Of note, further phase I clinical trials (NCT02269423, NCT02280408) (Table 1) employing high doses of rVSV-ZEBOV demonstrated dose-dependent induction of GP reactive antibody titers in all participants but only mild adverse events without further cases of arthritis (73).

A phase II/III clinical trial (NCT02378753) was initiated in Guinea in March 2015 assessing vaccine efficacy upon vaccination using one dose of 2 × 10^7^ PFU in a cluster randomization design with a ring vaccination approach (Table 1). Participants, including individuals at high risk, were assigned to clusters that were randomly subjected to immediate and delayed vaccination (21 days later). The study report demonstrated promising results (74, 75). No cases of Ebola virus disease with symptom onset at least 10 days after randomization were detectable in the immediate vaccination group, while 16 cases of Ebola virus disease from seven clusters occurred in the delayed vaccination group, demonstrating 100% vaccination efficacy. Of 43 serious events registered upon vaccination, only one was judged to be causally related to vaccination. Given these results, rVSV ZEBOV is currently the most promising candidate for a licensed vaccine against Ebola virus.

### Nucleic acid vaccines

Nucleic acid based technologies employ either antigen encoding plasmid DNA or RNA, as messenger RNA or viral replicons. Upon their cellular uptake and expression, nucleic acid encoded antigens can elicit humoral as well as cell-mediated immune responses. Both technologies are extremely versatile due to the ease of antigen manipulation they allow. The production of antigens in the target cells offers the advantage of mimicking protein synthesis during an infection, i.e., protein localizations such as presence in the plasma membrane and modifications such as glycosylation patterns can be formed with a high degree of faithfulness. Importantly, they support the delivery of any antigen of choice, regardless of whether it was derived from a virus, bacterium or parasite, supporting vaccine development against a wide array of pathogens. Since vaccine characteristics are independent of the encoded proteins, development of different vaccines can take place without the need to establish new production, purification and validation methods as well as manufacturing facilities. Hence, nucleic acid based technologies support fast and flexible vaccine development and production. Since all vaccines can be produced using the same basic components, manufacturing of several vaccines can take place in one established facility cutting both costs and time of vaccine production dramatically. Lastly, their synthesis mostly relies on chemically synthesized material, supporting large-scale production with relative ease.

#### DNA vaccines

##### Description

DNA vaccines are generated by insertion of a eukaryotic expression cassette encoding for the antigen(s) of choice into a bacteria-derived plasmid. The plasmid backbone generally contains elements that permit propagation and selection of the vector in *Escherichia coli*, i.e., an origin of replication that supports high yields of the plasmid during bacterial growth and a selectable marker, mostly the bacterial antibiotic resistance gene against Kanamycin, which allows stable inheritance of the vector. Since regulatory safety concerns have been raised against the presence of non-functional sequences, especially the antibiotic resistance marker, for human use, the marker has been replaced or removed in new generations of DNA vaccines (76). In addition, minimal DNA constructs devoid of a bacterial backbone, such as the semi-synthetic minicircle DNA (77) and the fully synthetic Doggybone™ (78), have been developed. The eukaryotic expression cassette is comprised of a 5′ promotor, typically derived from cytomegalovirus (CMV) that supports high transcription levels, the gene of interest and a 3′ polyadenylation (poly A) signal, required for nuclear export, translation and stability of the transcript mRNA, that is usually obtained from rabbit β-globin or bovine growth hormone genes (76).

##### Delivery of DNA vaccines

Research on DNA vaccines has started as early as the 1990s, where the most common route of administration was intramuscular (IM) or intradermal (ID) injection using a conventional needle. However, vaccination with a DNA vector alone generally leads to relatively low immunogenicity, especially in large animal models and humans. A factor that may play a role is the need for DNA vaccines to cross two cellular membranes, i.e., the plasma, as well as the nuclear membrane, in order to achieve protein expression. Of note, this does not hold true for RNA vaccines, which are translated upon crossing the plasma or endosomal membrane, respectively. Hence, additional methods have been developed that are able to enhance DNA uptake, expression and immunogenicity. These include various delivery devices such as gene gun, needle free injection devices (jet injection) and *in vivo* electroporation, which is among the most widely used and has been shown to yield promising results in both preclinical and clinical trials (79, 80). Furthermore, different formulations of DNA have been tested, i.e., encapsulation in lipid nanoparticles, containing cationic lipids and cholesterol, adsorption to polymers such as polyethyleneimine and adsorption or encapsulation in biodegradable nanoparticles, such as poly(lactic-co-glycolic acid) (PLGA) or chitosan (81). These methods are largely directed at improving the uptake of the DNA molecule into the cell and thus enhancing antigen expression. In addition, different approaches to modify and improve DNA mediated immune responses have been developed. For this, “molecular adjuvants” such as pattern recognition receptor (PRR) ligands and different cytokines, most commonly IL-12, are co-delivered with the encoded antigen and strategies to direct the antigen to certain cellular compartments or specifically target antigen presenting cells (APCs) to enhance immune responses have been established (82). In addition, DNA vaccines have successfully been employed for prime-boost regimen in combination with other vaccine technologies such as protein- or viral vector based vaccines.

##### Mode of action

Although a multitude of studies show that DNA vaccination is able to elicit both humoral and cellular immune responses, through activation of CD8^+^ cytotoxic and CD4^+^ helper T cells, respectively, the exact mechanism of action remains to be evaluated. Upon entry in the cell, DNA vaccines are sensed by a variety of innate immune receptors. While TLR9 is not critical for DNA vaccine efficacy, the STING/TBK1/IRF3 pathways and the AIM2 inflammasome are involved in DNA vaccine mode of action and other factors might additionally be involved (82). Early experiments testing bombardment with DNA coated gold particles delivered ID demonstrated transfection of both keratinocytes and professional APCs, i.e., Langerhans cells, explaining the source of both MHCI and MHCII restricted antigen recognition by CD8^+^ cytotoxic and CD4^+^ helper T cells, respectively (83). However, IM vaccination with DNA vectors mostly results in transfection of myocytes (84). Since several studies have established a role for bone marrow derived APCs in the activation of MHCI restricted CD8^+^ T cells upon DNA vaccination (85–87), the most likely mechanism in this scenario seems to be cross-priming and presentation of both MHCI and MHCII restricted antigens by professional APC upon phagocytosis of transfected somatic cells.

##### Advantages and disadvantages

As specified above, the use of nucleic acid based vaccines offers a number of advantages in different aspects of vaccine development and production. However, employing DNA as a basis for vaccination also implicates some disadvantages. A concern in this context is the long-term persistence of DNA plasmids upon injection. Indeed, DNA persistence was shown in various preclinical studies that demonstrated the presence of plasmid DNA for up to 2 years upon IM injection with low but detectable expression and immunogenicity in a mouse model (88). According to the FDA, DNA persistence is not generally evident at ectopic sites in biodistribution and persistence studies, but remains detectable at the injection sites for periods exceeding 60 days^8^. Especially in the context of this long-term persistence, the presence of foreign genetic information in the nucleus of transfected cells poses the additional risk of genomic integration into the host's chromosomes and the resulting threat of mutagenesis and oncogenesis. Despite negative results in several studies focusing on detection of DNA integration events upon IM injection in small animal models, genomic integration events were detectable following electroporation in mice (89, 90) demonstrating that integration represents a small risk that nevertheless needs to be considered in systems with enhanced DNA uptake. The FDA recommends integration studies to be included whenever plasmid DNA exceeding 30,000 copies per μg of host DNA persists in any tissue by study termination. The WHO advises integration studies as part of the preclinical safety program of DNA vaccines^9^. In addition, injection of bacterial DNA, sensed by the presence of unmethylated CpG motifs, has been associated with safety concerns, such as the generation of antibodies against the injected DNA. However, no anti-DNA antibodies have been detectable in mice, rats, rabbits or non-human primates (90). Potential expression of the antibiotic resistance marker in vaccinated organisms has likewise raised safety concerns that are met by the replacement of these markers in next generation DNA vaccines. Lastly, expression of cytokines or co-stimulatory molecules that are used to enhance DNA immunogenicity might lead to unintended adverse effects upon cytokine expression and release such as generalized immune suppression, chronic inflammation or autoimmunity. The WHO recommends monitoring the persistence of a cytokine expressing plasmid as well as appropriate preclinical models, such as animal models responsive to the respective human cytokine to ensure vaccine safety.

##### DNA vaccines in potential pandemic settings

Since the first experiments in the 1990 (91), DNA vaccines have been employed for vaccine development up to clinical trials against a large variety of human pathogens such as HIV, influenza virus, malaria, hepatitis B virus, respiratory syncytial and herpes simplex virus. No DNA based vaccine is licensed for human use as yet, but several DNA based vaccines have been licensed for veterinary applications, such as an equine vaccine against West Nile Virus. Given their high degree of versatility, DNA vaccines have been tested for their efficacy to protect against recent pandemic threats including HIV, MERS, Ebola, and Zika, some of which will be discussed in more detail below.

The first effective vaccines against **Ebolaviruses** developed in preclinical experiments employed DNA vector based antigen expression. These approaches relied on expression of the viral glycoprotein (GP), to induce neutralizing antibodies as well as nucleoprotein (NP) as a target for antibody as well as T cell responses. Induction of both humoral and T cell-mediated immunity as well as protective efficacy against rodent adapted viral strains was demonstrated in guinea pigs and mice, upon vaccination with DNA encoding for GP and NP using intramuscular injection or intradermal delivery using a gene gun, respectively (92, 93). Later studies established protection induced by a trivalent DNA vaccine encoding for GP of two Ebolaviruses and a Marburgvirus (94) and protection from lethal challenge against an Ebolavirus [Ebola virus (EBOV)] upon DNA vaccination in combination with adenoviral vectors in non-human primates (55). Having a set of promising preclinical data established, the first phase I clinical trial (NCT00072605) using a DNA vaccine against Ebola was started in 2003, well before the Ebola crisis in 2014 (95) (Table 2). This study employed a trivalent DNA vaccine consisting of plasmids encoding for transmembrane-deleted forms of GP derived from two Ebolaviruses as well as NP produced by Vical Inc. Results demonstrated safety and tolerability of this vaccine as well as specific antibody responses to at least one of the three antigens in all subjects. However, no detectable virus neutralizing responses were elicited in this trial. A further phase I clinical trial (NCT00605514) conducted in 2008–2009 (96) employed wildtype GP constructs that had been found to elicit superior responses over transmembrane deletions of GP in the context of adenoviral delivery in NHPs (57) (Table 2). Two different DNA vaccines encoding for GPs of two species of Ebolavirus (produced by the VRC/NIAID Vaccine Pilot Plant, operated by Leidos) or Marburg Marburgvirus (MARV) GP (produced by Althea Technologies), respectively, were administered. This study confirmed safety of both DNA vaccines. 80% of subjects were found to elicit specific antibody responses against one of the GPs. Given the reassuring safety profile, a phase Ib study (NCT00997607) was conducted in Uganda in 2009 (97) (Table 2). Both vaccines were well tolerated but immune responses remained poor with around 50% and 30% of the subjects eliciting antibody responses against the Ebolavirus and MARV components, respectively. Overall, results of these early generations of DNA based vaccines were somewhat discouraging. However, efforts were renewed using improved DNA technologies, upon the outbreak in 2014. Inovio is developing and testing their GP encoding DNA vaccine candidate INO-4212 (a combination of two DNA vaccines, i.e., INO-4201 and INO-4202, encoding for GP derived from a pre-2013 and a current viral isolate, respectively). Proving the versatility and speed of the approach, a clinical trial was initiated in early 2015 (NCT02464670) (Table 2). The study assesses vaccine safety, tolerability, and immunogenicity of the components with and without an IL-12 encoding plasmid (INO-9012). Preliminary results have shown a favorable safety profile; ~90% of the participants generated an Ebola-specific antibody immune response.

**Table 2:** Clinical trials employing DNA vaccines in pandemic settings.

**Study start**	***N***	**Vaccine and delivery**	**Outcome**
**NCT00072605**		**EBOLA**	**Phase I**
Oct 2003	27	**DNA**, trivalent; NF inj.dev. IM 2–8 mg in week 0, 4, and 8 Antigens: - GPΔTM EBOV - GPΔTM SUDV - NP	**Safety:** Acceptable safety profile **Immunogenicity:** - Specific antibody response to at least 1/3 antigens in all subjects - Specific CD8^+^ T cell responses in 30% (6/20) subjects. - No detectable virus neutralizing responses
**NCT00605514**		**EBOLA**	**Phase I**
Jan 2008	20	**DNA**, mono or bivalent; NF inj.dev. IM 4 mg in week 0, 4, 8; (32) Antigens: - GP MARV - GP EBOV + GP SUDV	**Safety:** Acceptable safety profile **Immunogenicity:** - Specific antibody responses against one of the GPs at week 12 in 80% of subjects - CD8^+^ T cell responses in some of the subjects
**NCT00997607**		**EBOLA**	**Phase Ib**
Feb 2010	108	**DNA**, mono or bivalent; NF inj.dev. IM 4 mg in week 0, 4, 8 Antigens: - GP MARV - GP EBOV + GP SUDV	**Safety:** Acceptable safety profile **Immunogenicity:** Specific antibody responses in 30% (MARV) and 50% (EBOV or SUDV) of subjects Antibody titers to near baseline levels by w 44 post vaccination
**NCT02464670**		**EBOLA**	**Phase I**
May 2015	240	**DNA**, mono-, bi- or trivalent; IM or ID + EP in 2 or 3 doses 0.8–4 mg GP; 0.2–1 mg IL12 Antigen: - GP EBOV pre 2013 - and/or GP EBOV 2014 - and IL-12 in trivalent vaccine	**Safety:** Acceptable safety profile **Immunogenicity:** Specific antibody responses in 88% (50/57) (IM) and 95% (119/122) (ID) of participants
**NCT00709800 and NCT00694213**		**INFLUENZA H5N1**	**Phase I**
Aug 2007	103	**DNA**, mono- or trivalent; needle or NF inj.dev. IM 0.1–1 mg in week 0, 3 Antigen: - HA of A/Vietnam/1203/04 - HA + NP + M2	**Safety:** Acceptable safety profile **Immunogenicity:** - HI titers ≥40, in 47- 67% (HA only) and 0- 20% (HA + NP + M2) of participants, peak at d56 - H5-specific T cell responses in 75–100% (HA only) and 50–57% % (HA + NP + M2) of subjects - Responses against HA unaffected by injection method
**NCT00973895**		**INFLUENZA H1N1**	**Phase I**
Aug 2009	20	**DNA**, monovalent; NF inj.dev. IM 4 mg in week 0, 4, 8 Antigen: HA of A/California/04/2009	**Safety:** Acceptable safety profile **Immunogenicity:** - HI titers ≥40 in 30% (6/20) of DNA vaccinated subjects - DNA + licensed vaccine HI titers ≥40 in 72% (13/18) - T cell responses in 25% (5/20) of subjects
**NCT02809443 (NCT02887482)**		**ZIKA**	**Phase I**
July 2016 (Aug 2016)	40 (160)	**DNA**, monovalent; ID + EP 1 or 2 mg in week 0, 4, 12 Antigen: Consensus prM-E; IgE SP; removed glycosylation site	**Safety:** Acceptable safety profile (NCT02809443) **Immunogenicity (preliminary results NCT02809443):** - VNTs in 62% of the participants (Vero cell assay) - Protection of 92% (103/112) of mice by passive serum transfer in challenge model (IFN α/β receptor knockout)
**NCT02840487; NCT02996461**		**ZIKA**	**Phase I/Ib**
Aug 2016 Dec 2016	125	**DNA**, monovalent; needle or NF inj.dev. IM 4 mg in 2 or 3 doses Antigen: - prM-E; JEV SP (VRC5283) - prM-E; JEV SP and S/TM (VRC5288)	**Safety:** Acceptable safety profile **Immunogenicity:** - Humoral and T cell responses induced - VNTs in 60%−100% of subjects 4 w after the final vaccination - Best responses in VRC5283: Antibody responses in 100% (14/14) of participants in NF inj, in split doses group; best VNT and T cell responses
**NCT03110770**		**ZIKA**	**Phase II**
Mar 2017	2500	**DNA**, monovalent; NF inj.dev. IM in 3 doses 4 mg or 8 mg in 2 or 4 inj. Antigen: - prM-E; JEV SP (VRC5283)	Results pending, estimated study completion date Jan 2020

*This table exclusively lists clinical trials discussed in the text. EBOV, Ebola virus; EP, electroporation; GP, glycoprotein; GPΔTM, glycoprotein delta transmembrane domain; HA, hemagglutinin influenza; HI, hemagglutination inhibition; ID, intradermal; IL-12, interleukin 12; IM, intramuscular; JEV, Japanese encephalitis virus; M2, ion channel protein influenza; MARV, Marburg virus; N, number of study participants; NF inj.dev, needle free injection device; NP, nucleoprotein influenza; prM-E, preMembrane-Envelope; SUDV, Sudan virus; VNT, virus neutralization titer; SP, signal peptide; S/TM, stem and transmembrane regions*.

A large number of preclinical and clinical studies have assessed the ability of DNA vaccines to mediated protection against **influenza** viruses, either alone or as part of prime boost strategies. These vaccines mainly rely on plasmid based expression of hemagglutinin (HA), one of the viral surface antigens and the main target for neutralizing antibodies against influenza. In terms of pandemic preparedness in DNA only vaccination strategies, Vical Inc. has developed and tested a vaccine that targets the highly pathogenic avian H5N1 influenza endemic in poultry. Its ability to cross the species barrier, that was first discovered in 1997 and caused rising numbers of human infections between 2003 and 2008, renders this virus a high pathogenic risk. So far, the virus is not able to spread efficiently and sustainably from human to human but H5N1 bird to human infections have caused the death of 453 people worldwide until 2017.^10^ DNA vaccines expressing HA of the viral strain A/Vietnam/1203/04 were either employed alone or in combination with the conserved nucleoprotein (NP) and ion channel protein (M2) derived from different subtypes as targets of T cell responses. NP and M2 had previously been shown to protect mice against lethal challenge in the absence of an HA component (98). Clinical trials testing DNA vaccines in combination with the lipid-based adjuvant Vaxfectin® were initiated in 2007 after protective efficacy was demonstrated in preclinical studies in mice and ferrets (99) (NCT00709800 and NCT00694213) (Table 2). Vaccines were found to be well tolerated and HI titers ≥40, the correlate of protection, were elicited in a maximum of 67 and 20% in HA only and trivalent groups, respectively.

Upon emergence of a novel H1N1 influenza that originated in pigs and became pandemic in humans in spring 2009 (100), efforts were made for the accelerated development of a vaccine. A clinical trial (NCT00973895) was initiated by August 2009 using a DNA based approach encoding hemagglutinin protein of A/California/04/2009(H1N1pdm09) whose GMP production was finalized 2 months before licensed monovalent influenza vaccines became available (101) (Table 2). However, 4 weeks after the last vaccination, only 30% of subjects had developed positive HI responses that increased to 72%, 4 weeks after boosting with a licensed monovalent influenza vaccine. Based on results gained at this point, the ability for fast manufacturing of a large number of doses could support the use of DNA-based vaccines for controlling a potential influenza pandemic by employing DNA as an initial priming agent, followed by boosting with conventional influenza vaccines upon availability.

DNA based vaccines were among the first to proceed to clinical trials upon the **Zika** crisis in 2016. Leveraging knowledge generated in the context of other flaviviruses, these approaches rely on the expression of the precursor membrane and envelope (Env) (prM-E) proteins which are known to form subviral particles with Env being the target of virus neutralizing antibodies. The first approach developed by Inovio employed a consensus prM-E derived from African and more recent Asian and American strains modified to contain an IgE signal peptide with a putative glycosylation site removed (GLS-5700) (102). This vaccine was shown to be immunogenic and protective in a mouse model upon IM vaccination followed by electroporation. Passive transfer experiments of vaccine-induced sera in an interferon (IFN) α/β receptor knockout mice demonstrated correlation of antibody levels with protection. Furthermore, the induction of virus antibodies and T cell responses upon ID vaccination followed by electroporation was shown in NHPs. Based on these results, two phase I clinical studies were initiated, one in flavivirus-naive individuals (NCT02809443) that was started in July 2016 and the other one in dengue virus seropositive subjects (NCT02887482) which began in August 2016 (Table 2). Preliminary results from NCT02809443 (103) demonstrated that the vaccine was well-tolerated and induced neutralizing antibodies in 62% of the participants.

A preclinical study published in October 2016 demonstrated the induction of neutralizing antibodies and protection from challenge infection in 17 of 18 NHPs upon two IM vaccinations using a needle free injection device. This study employed two different prM-E constructs based on the sequence of French Polynesian and early Brazilian ZIKV isolates in which the Zika prM signal sequence alone (VRC5283) or in combination with the stem and transmembrane regions (VRC5288) were exchanged with the corresponding sequences from Japanese encephalitis virus (JEV). Both vaccine candidates are evaluated in clinical studies by The Vaccine Research Center (VRC), National Institute of Allergy and Infectious Diseases (NIAID) (Table 2). Clinical trials testing VRC5288 (NCT 02840487) and VRC5283 (NCT02996461) were initiated in August 2016 and December 2016, respectively. The results of these phase I studies were published in the Lancet in December 2017 (104). Both trials showed that vaccinations were safe and well tolerated and induced both humoral and T cell responses. Positive neutralizing antibody responses ranging from 60 to 100% were detected 4 weeks after the final vaccination; VRC5283, in agreement with preclinical studies, yielded better responses than VRC5288.

Both DNA based approaches for the development of an effective Zika vaccine appeared safe for human use and yielded promising results. Importantly, they were initiated within months after sequences became available, highlighting the versatility and speed provided by DNA vaccine platforms.

#### RNA vaccines

##### Description

mRNA is an intermediate carrier of genetic information used as template for endogenous protein production in the vaccinated subject. Two major types of RNA have been utilized as prophylactic vaccines against pathogens that cause infectious diseases:

1. Non-replicating mRNA
2. Self-amplifying mRNA

**Non-replicating mRNA** contains the sequence of the antigen of choice flanked by 5′ and 3′ untranslated regions (UTRs). The advantages of using non-replicating mRNA vaccines compared to self-amplifying mRNA are rooted in the simplicity of the construct, the small size of the RNA, and the absence of any additional encoded proteins that could induce unintended immune responses (105). The design of optimized, efficiently translated mRNA for use as a vaccine has been reviewed previously (105–107). Briefly, conventional non-replicating mRNA is obtained by *in vitro* transcription of a cDNA template, typically plasmid DNA (pDNA) produced in *E. coli*. The pDNA template is linearized using restriction enzymes and is transcribed *in vitro* into mRNA in a mixture containing recombinant phage DNA-dependent RNA polymerase (typically derived from T7 or T3 or Sp6 phage) and nucleoside triphosphates (NTPs) (108). Upon purification, usually via FPLC or HPLC to remove any remaining product related impurities such as reaction components (i.e., enzymes, free NTPs, residual pDNA) or abortive transcriptional byproducts, a pure single mRNA product is obtained (109). Notably, purification of *in vitro* transcribed mRNA seems to be crucial for the amount of immunogen produced in target cells as demonstrated by up to 1,000-fold increased protein production in primary human DCs transfected with HPLC purified compared to unpurified mRNA (110). The *in vitro* transcribed mRNA product contains a protein-encoding open reading frame (ORF) flanked by elements essential for the function of mature eukaryotic mRNA: a cap structure, joined to the 5′ and a poly(A) tail at the 3′ end, as well as 5′ and a 3′ untranslated regions (UTR) (111–113). The 5′ cap is vital for the creation of stable mature mRNA and increases protein translation via binding to eukaryotic translation initiation factor 4E (111, 114). The 5′ cap can be added either during the transcription by inclusion of a cap analog or anti-reverse cap (ARCA) in the reaction (115), or subsequently, using the vaccinia virus capping complex (116). The UTRs, which can be of eukaryotic or viral origin, increase the half-life, and stability of the mRNA, resulting in higher expression of the protein (117–120). The poly A tail of an optimal length is an essential regulatory element to enhance translation and can be either encoded into the DNA template or alternatively added enzymatically post transcription (111, 121, 122). The sequence of the ORF can be optimized using either enrichment of the GC content (123–125) or by replacement of rare codons by frequently used synonymous codons leading to increased protein production from mRNA (126). Utilization of chemically modified nucleosides can decrease innate immune activation and increase translation of the mRNA (127).

**Self-ampifying mRNA vaccines** are most commonly based on the alphavirus genome [reviewed in detail in (128–130)], from which the genes encoding the structural protein have been replaced with the antigen of choice. Despite these gene deletions, the viral RNA is replicated and transcribed by the viral RNA polymerase. The full length mRNA of the self-amplifying mRNA vaccines is substantially larger (~9–10 kb for alphavirus systems) than in non-replicating mRNA vaccines, but contains the same essential elements such as a cap, 5′ and 3′ UTRs, and poly A tail (128). Of note, lower yields and increased occurrence of abortive constructs as a consequence of the large size of these vaccines pose challenges to vaccine production, that make manufacturing processes more difficult compared to non-replicating mRNA vaccines. The additional mRNA contains a sub-genomic promoter and a large ORF encoding for non-structural proteins which, following delivery of the vaccine into the cytosol, are transcribed in four functional components (nsP1, nsP2, nsP3, and nsp4) by the encoded RNA-dependent RNA polymerase (RDRP) (131). RDRP than produces a negative-sense copy of the genome which serves as a template for two positive-strand RNA molecules: the genomic mRNA and a shorter sub-genomic mRNA. This sub-genomic mRNA is transcribed at very high levels, allowing the amplification of mRNA encoding the antigen of choice. Hence, any genetic information encoded by the self-amplifying mRNA vaccine will be amplified many times, resulting in high levels of antigen expression from relatively low doses of the vaccine, which is an appealing attribute of self-amplifying mRNA vaccines compared to non-replicating mRNA vaccines (132). Upon injection in mice, LNP-formulated self-amplifying mRNA encoding firefly luciferase induced protein expression lasting almost two months upon IM delivery (130), while luciferase expression from protamine-formulated, non-replicating mRNA administered ID was usually only detected for several days (133). However, potential interactions between the host and the encoded alphaviral non-structural proteins necessitate further investigation.

Self-amplifying mRNA is most commonly delivered with synthetic delivery vehicles as discussed below. An alternative method is packaging and delivery in virus-like replicon particles (VRPs) produced by a helper cell line that provides the capsid and glycoprotein genes in trans (134). While the lack of structural protein genes contained in VRPs prevents production of further viral particles and cell-to-cell spread, VRPs are capable of infecting cells and expressing the antigen of choice *in vitro* and *in vivo*. Although both preclinical and clinical data for the VRPs are promising, this technology requires the use of electroporation of the genetic material into cell culture cells during the manufacturing process. Although electroporation has been successfully employed under GMP conditions at a scale sufficient to provide material for a phase I study, cost-effective production at industrial scale may be challenging. In addition, there are some safety concerns associated with VRPs, since recombination or co-packaging of replicon and helper RNAs VRPs during their production in cells containing both replicon and helper RNAs could lead to the generation of infectious viruses.

##### Delivery of mRNA vaccines

In order to act as a vaccine, exogenous mRNA has to enter the cytoplasm where protein expression can take place. In this step, the plasma or endosomal lipid membrane represents a barrier the mRNA vaccine has to cross as efficiently as possible. In addition, the induction of an effective immune response requires stimulation of the innate immune system by the mRNA vaccine. While mRNA has some intrinsic innate stimulation function (see below), this effect can be increased by different ways of mRNA formulation. Hence, several methods to increase both cell delivery and adjuvanticity of mRNA vaccines have been developed.

Immunization can take place via direct injection of **naked mRNA**, especially via routes which lead to effective targeting of APCs, such as intradermal (135–137) and intranodal (138–140) administration. However, when delivered IM, humoral and cellular immune responses induced by naked mRNA remain low compared to LNP-formulated mRNA (141).

Physical delivery methods of mRNA vaccines that likely increase vaccine release into the cytoplasm have been shown to induce immune responses in mice upon administration of non-replicating mRNA and self-amplifying mRNA using a gene gun and *in vivo* electroporation, respectively (142–146).

A more commonly used strategy to increase expression and immunogenicity is the delivery of mRNA in complex with additional components. Among the first approaches was a format, whose two components, free and **protamine-complexed** mRNA (a small arginine-rich nuclear protein that stabilizes nucleic acids), provide both strong antigen expression and immunostimulation (147–150). This vaccine format has proved to be immunogenic and capable of inducting protection against lethal challenge infections with influenza or rabies virus in several animal models (124, 151). Using this format, CV7201, a candidate vaccine against rabies, was investigated as the first ever prophylactic mRNA-based vaccine in healthy human volunteers. The subjects received 80–640 μg of the mRNA vaccine three times by conventional needle-based injection or needle-free injection devices via the intradermal (ID) or intramuscular (IM) route. The vaccine was generally safe with a reasonable tolerability profile and led to the induction of neutralizing antibody titers at levels of 0·5 IU/mL or higher (as the correlate of protection) in 71% of subjects who had received ID injections of 80 or 160 μg mRNA vaccines by needle-free intradermal injection, while needle-based injection was ineffective (152). Antibody responses waned one year after first vaccination but could be boosted to 0·5 IU/mL or higher in 57% of subjects using 80 μg of mRNA delivered ID with a needle free injection device, indicating the induction of B cell memory responses. Although the mRNA vaccine candidate was able to induce antibody responses, further improvements to increase the magnitude and longevity of the immune responses are imperative for the development of an effective vaccine.

The efficacy of mRNA vaccines can benefit significantly from complexing agents such as **lipid- and polymer-based nanoparticles** which enhance uptake by cells and improve delivery to the translation machinery in the cytoplasm. Although commercially available cationic lipids and polymers [e.g., TransIT-mRNA (Micrus Bio LLC) or Lipofectamine (Invitrogen)] are efficient transfection reagents for mRNA in cell lines and primary cells (110, 127) their use for *in vivo* mRNA delivery is limited due to high toxicity and low efficacy of transfection. Safer and more effective complexing reagents which were discussed in detail in some recent reviews (153–156) have been designed in the past few years, leading to the expansion of the field for prophylactic use and the development of more potent and versatile mRNA vaccines. Currently, lipid nanoparticles (LNPs) are the most promising and frequently used class of agents for *in vivo* delivery of mRNA vaccines. LNPs have been intensively studies in the context of siRNA (157) and are well tolerated compared to other non-viral delivery system. Most LNPs rely on ionizable amino lipids which complex the negatively charged mRNA, support assembly into 70–100 nm sized particles and promote escape of the mRNA from endosomal compartments into the cytoplasm where the mRNA can be translated. In addition to ionizable amino lipids, phospholipids, cholesterol and lipid-anchored polyethylene glycol (PEG) are the most commonly used components for LNP formulations. Cholesterol acts as a stabilizing element and plays an important role in the transfection of cells. Lipid-anchored PEG preferentially deposits on the LNP surface, where it can act as a barrier which sterically stabilizes the LNP and reduces non-specific binding to proteins increasing the half-life of the LNPs. Furthermore, the surface of an LNP can be decorated with specific targeting entities which direct the vaccine to certain tissues or cells, such as professional APCs, thereby facilitating the uptake of the mRNA vaccine by the desired type of immune cell and eventually leading to an enhanced immune response against the antigen of choice. Several studies demonstrated that LNPs are effective agents for *in vivo* delivery of non-replicating and self-amplifying mRNA vaccines (130, 141, 158, 159).

In addition to formulation, the **route of mRNA administration** has a crucial impact on the quality and strength of the induced immune response. LNP-mRNA delivered intravenously (IV) primarily targets the liver (160), while ID and IM delivery generally show more prolonged expression of the antigen of choice at the injection site (141, 159, 161). A study comparing different routes of administration of LNP-formulated mRNA coding for luciferase showed that the total amount of protein produced was largest for IV administration, while duration of luciferase expression was the longest for ID followed by IM injection (161). **Intradermal (ID) injection** delivers mRNA vaccines directly into the skin, an organ densely populated with professional APCs such as Langerhans cells in the epidermis and various dendritic cells (DC) subtypes in the dermis. The ID route of administration has been shown to effectively induce a balanced immune response including antibodies as well as Th1 type and cytotoxic T cells for mRNA vaccines formulated in protamine or LNP (124, 150, 158).

**The intramuscular (IM) injection** of vaccines is the most often practiced route of administration in humans. Since this route of vaccination is simple to carry out and does not require much training for its implementation, it may be the preferred route of administration by the physicians carrying out immunization in regions affected by a pandemic. However, the need for educated personnel to vaccinate people might represent a limiting factor in the face of a pandemic. The induction of strong immune responses after IM injection of mRNA represents a high hurdle, due to lack of co-stimulatory molecules and optimal antigen presentation on muscle cells and low infiltration of the muscle tissue by immune cells. Thus, potent IM mRNA vaccines must allow high antigen expression and presentation and simultaneously induce strong immunostimulatory signals to recruit immune cells to the injection site. The IM administration of non-replicating nucleoside-modified mRNA-LNP vaccines against the Zika virus, as well as influenza A H10N8 and H7N9 viruses proved to be immunogenic and provide protection in preclinical studies in mice, ferrets and NHPs (159, 162, 163). Single IM immunization of NHPs with LNP-formulated mRNAs encoding rabies or influenza antigens induced protective antibody titers, which could be boosted and remained stable during an observation period of up to one year (141).

##### Mode of action

Exogenous mRNA is immunostimulatory, as it is recognized by a variety of cell surface, endosomal and cytosolic innate immune receptors. Mammalian cells can sense foreign RNA via PRRs such as TLR3, TLR7 and TLR8 located in the endosomes and RIG-I, MDA-5 and PKR located in the cytoplasm as well as NLRP3 and NOD2 (164). Activation of the PRRs by mRNA vaccines results in a robust innate immune response including production of chemokines and cytokines such as IL-12 and TNF at the inoculation site (165), which are innate factors crucial for the induction of an effective adaptive immune response against the encoded antigen. ID immunization with mRNA vaccines upregulates the expression of chemokines including the CXCR3-ligands CXCL9, CXCL10, and CXCL11, that recruit innate immune cells such as DCs and macrophages, to the site of injection (165). Kowalczyk et al. showed that the in the skin, protamine-formulated non-replicating sequence optimized mRNA vaccines are taken up by both non-leukocytic and leukocytic cells, the latter being mostly represented by APCs (150). mRNA was then transported to the draining lymph nodes (dLNs) by migratory dendritic cells. Moreover, the encoded protein was expressed and efficiently presented by APCs within the dLNs as shown by T cell proliferation and immune cell activation, followed by the induction of the adaptive immunity. Importantly, the immunostimulation was limited to the injection site and lymphoid organs as no proinflammatory cytokines were detected in the serum of the immunized mice. Lazzaro et al. demonstrated that CD8^+^ T-cell priming is restricted to bone-marrow-derived APCs and may involve antigen transfer from myocytes suggesting cross-priming as the prevalent mechanism upon IM injection of self-amplifying mRNA vaccines in mice (166). In a recent publication, Lutz et al. provided first mechanistic insights into the mode of action of LNP-formulated non-replicating sequence optimized mRNA vaccines, demonstrating a strong activation of the innate immune response at the injection site and in the dLNs in mice. IM injection of LNP-formulated mRNA vaccine resulted in spontaneous uptake of the mRNA by cells surrounding the injection site and strong expression inside transiently transfected cells, including resident professional APCs, neutrophils and non-leukocytic cells (141). Interestingly, similar observations were published using LNP-formulated non-replicating mRNA vaccines containing modified nucleotides which induced rapid and local infiltration of neutrophils, monocytes, and DCs to the site of administration and the dLNs in injected NHPs (167). While these cells efficiently internalized LNPs, mainly monocytes and DCs translated the mRNA and up-regulated key co-stimulatory receptors (CD80 and CD86). This coincided with upregulation of type I IFN-inducible genes, including Mx1 and CXCL10. The innate immune activation was transient and resulted in priming of antigen-specific CD4^+^ T cells exclusively in the vaccine-draining LNs. The data demonstrate that mRNA-based vaccines induce type-I IFN-polarized innate immunity and, when combined with antigen production by APCs, lead to generation of potent vaccine-specific responses. Professional APCs, with DCs likely being the most relevant cell type for mRNA vaccines, play a critical role in antigen processing and presentation to elicit an immune response against specific antigens. The transfected DCs express the mRNA-encoded antigen in the native form. Expressed proteins are subsequently processed into antigenic peptides and are presented on MHC class I and MHC class II molecules along with co-stimulatory signals to CD8^+^ and CD4^+^ T cells, respectively. Antigen expressed in the correctly folded native form can be recognized by B cells that in response produce antibodies against the antigen. A study in NHPs investigating the immunological events leading to antibody responses elicited by a modified non-replicating mRNA encoding influenza A H10 HA encapsulated in LNPs showed that, while both ID and IM administration induced titers considered to be protective, ID delivery generated this response more rapidly (168). Circulating influenza H10-specific memory B cells expanded after each of the two immunizations, along with a transient appearance of plasmablasts. The memory B cell pool waned over time but remained detectable throughout the 25-week study. Following immunization, H10-specific plasma cells (PCs) were detected in the bone marrow and persisted throughout the 25 week observation period with a more profound decline detected in IM group compared to the ID group by the end of the study. Germinal centers were formed in vaccine-draining lymph nodes along with an increase in circulating H10-specific ICOS^+^ PD-1^+^ CXCR3^+^ T follicular helper cells, a population shown to correlate with high avidity antibody responses after seasonal influenza vaccination in humans. In addition, a non-replicating sequence optimized mRNA vaccine induced long-lived functional antibody responses against HA of influenza A H1N1pdm in NHPs which persisted for one year (141). These results indicate that non-replicating mRNA vaccines potently induce an immunological repertoire associated with the generation of high magnitude long-lived antibodies.

##### Advantages and disadvantages

Although injection of naked mRNA via the ID or intranodal (135–140) route has been reported to induce immune responses, mRNA alone is not applicable for broad use as a prophylactic vaccine. Because of the omnipresence of extracellular ribonucleases which catalytically hydrolyze RNA, unprotected “naked” mRNA is highly unstable under physiological conditions and due to the hydrophilicity and strong net negative charge of RNA not taken up efficiently by cells after application *in vivo*. However, this challenge has been overcome by complexing of mRNA with highly efficient carriers such as new generations of LNP described above, which protect the mRNA from ribonucleases and allow prolonged *in vivo* expression of the antigen of choice leading to the generation of potent humoral and cellular immune responses following *in vivo* administration.

Activation of the innate immune response by RNA vaccines is potentially a double-edged sword. While systemic type I IFN produced in response to the activation of PRRs can facilitate the adaptive immune response, it can lead to phosphorylation of eukaryotic translation initiation factor 2α (eiF2α) which results in a slowdown and eventually inhibition of protein translation. Pepini et al. report that a self-amplifying mRNA vaccine elicits an inflammatory response within a few hours indicated by the upregulation of several IFN-stimulated genes and that antigen expression and immunogenicity were both enhanced in the absence of IFN-α/β signaling, suggesting that reduction of early type I IFN responses could improve RNA vaccine potency (169). Several approaches have been described which aim at overcoming the stalled translation and increased degradation of mRNA induced by the activation of the type I interferon pathway. One such approach is the use of naturally occurring modified nucleotides to suppress activation of the innate receptor-mediated responses. Kariko and others found that, compared to unmodified mRNA, nucleoside-modified mRNA was translated more efficiently *in vitro* in primary DCs and *in vivo* in mice (127, 170). The second approach developed by CureVac AG is based on the optimization of the nucleotide sequence, and hence the codon usage, relying exclusively on unmodified nucleotides which affects both mRNA stability and immunogenicity. As shown by Thess and colleagues, sequence-optimized, unmodified mRNA led to higher protein expression *in vitro* in HeLa cells and *in vivo* in mice than the respective mRNA containing modified nucleosides (123). However, it remains to be determined which approach, modified or unmodified mRNA, provides a better basis for prophylactic vaccines in humans.

In recent human clinical studies, mild to moderate and in rare cases severe local and systemic reactions were reported for different mRNA platforms (152, 159). Future studies in suitable animal models should carefully evaluate the distribution of the mRNA, expression of the encoded antigen in distant organs, potential safety risks, including local and systemic effects, toxic effects of new delivery systems, as well as the induction of self-reactive antibodies in humans.

mRNA vaccines, like DNA vaccines, are able to induce both humoral and cellular immune responses, encode any antigen of choice and allow a high degree of adaptability. In terms of manufacturing, both platforms allow production of different vaccines using the same established production process and facility. However, since the production process of mRNA is based on *in vitro* systems and does not require amplification in bacteria or cell cultures, manufacturing of mRNA vaccines is a comparably short and simple to monitor process. As mRNA vaccines do not interact with the host-cell DNA, they avoid the potential risk of genomic integration posed by DNA-based vaccines. Since mRNA vaccines represent a minimal vector containing the ORF encoding the antigen of choice flanked by specific regulatory elements, they do not induce anti-vector immunity as observed for certain viral vector-based platforms (171, 172) and therefore can be administered multiple times. Furthermore, mRNA vaccines can be administered by different routes using conventional needle-based injections and, unlike DNA vaccines, they do not require any additional administration device such as gene gun or electroporation. Therefore, mRNA vaccines offer a flexible one-for-all large-scale, rapid and cost-effective manufacturing process with fast turnaround time. This is vital when facing a pandemic threat requiring a rapid response platform capable of producing protective vaccines in the short time-frame necessary to protect at-risk populations and have an early impact on the progression of an outbreak.

##### RNA vaccines in potential pandemic settings

An increasing number of preclinical studies have shown promising results for both self-amplifying and non-replicating mRNA vaccines to confer protection against various pathogens, including those with pandemic potential (162, 173–176).

**Self-amplifying mRNA vaccines** encoding various influenza antigens complexed with LNP or oil-in-water cationic nanoemulsions (CNE) were immunogenic in ferrets, facilitating containment of viral replication in the upper respiratory tract upon influenza infection and conferred protection against homologous and heterosubtypic viral challenge in mice (173, 177, 178). A self-amplifying mRNA vaccine encoding an HIV-1 clade C envelope glycoprotein formulated in CNE, induced potent cellular as well as binding and neutralizing antibody responses in NHPs (179). RNA replicons encoding the glycoprotein complex of the Lassa virus encapsulated into VRP particles were immunogenic and protective in mice and resulted in induction of cross-reactive multifunctional T cell responses (176). Chahal et al. demonstrated in a mouse model that a modified dendrimer nanoparticle (MDNP)-based RNA replicon vaccine platform provides protection against lethal influenza and Ebola virus infections and elicits antibody and CD8^+^ T cell responses against Zika virus (180, 181). However, so far, self-amplifying mRNA vaccines have not been tested in clinical studies and their safety, tolerability and efficacy in humans has yet to be proven.

A variety of preclinical studies have demonstrated the ability of **non-replicating mRNA** vaccines to induce immune responses and confer protection against pathogens with pandemic potential such as ZIKV, EBOV and influenza. Importantly, some of these approaches are currently being tested in clinical trials. Pardi et al. demonstrated that ID immunization with LNP-encapsulated modified mRNA encoding the prME glycoproteins of **ZIKV** elicited potent and durable neutralizing antibody responses that were protective in mice and NHPs (158). A subsequent study by Richner et al. showed that IM administration of a similarly designed ZIKV vaccine resulted in high levels of neutralizing antibody titers that were protective, conferred sterilizing immunity and restricted *in utero* transmission of ZIKV in mice (162, 163). A Phase I/II, randomized, placebo-controlled, dose-ranging study of this ZIKV mRNA vaccine (mRNA-1325) was initiated in December 2016 with an estimated primary completion date in September 2018 (NCT03014089) (Table 3).

**Table 3:** Clinical trials employing RNA vaccines in pandemic settings.

**Study start**	***N***	**Vaccine and delivery**	**Outcome**
**NCT03014089**		**ZIKA**	**Phase I/II**
Dec 2016	90	**mRNA 1325**, modified nucleotides; LNP-formulated, Antigen: prM-E polyprotein	Results pending; estimated primary completion date in Sept 2018
**NCT03076385**		**INFLUENZA H10N8**	**Phase I**
Dec 2015	201	**mRNA 1851**, modified nucleotides; LNP-formulated, Antigen: HA of H10N8 A/Jiangxi-Donghu/346/2013	Interim results published for 100 μg IM (*N* = 23) vs. placebo (*N* = 8) **Safety:** acceptable safety profile **Immunogenicity:** - HI titers ≥40 in 100% (23/23) of subjects at day 43 - MN ≥20 in 87% (20/23) at day 43
**NCT03345043**		**INFLUENZA H7N9**	**Phase I**
May 2016	156	**mRNA 1440**, modified nucleotides; LNP-formulated, Antigen: HA of H7N9 A/Anhui/1/2013	Results pending; estimated primary completion date in Sept 2018
**NCT03325075**		**CHIKUNGUNYA**	**Phase I**
Aug 2017	60	**mRNA 1388**, modified nucleotides; LNP-formulated Antigen: structural polyprotein	Results pending; estimated primary completion date in Sept 2019

*This table exclusively lists clinical trials discussed in the text; prM-E, preMembrane-Envelope; HA, Hemagglutinin; HI, hemagglutination inhibition; MN, microneutralization titers; N, number of study participants; IM, intramuscular; ID, intradermal; LNP, lipid nanoparticle*.

In the context of **Ebolavirus** vaccines, LNP-encapsulated modified mRNA encoding EBOV GP delivered IM was shown to induce EBOV-specific IgG and neutralizing antibody responses and protected guinea pigs against lethal infection and signs of clinical illness (175). However, no clinical studies employing mRNA vaccines in the context of Ebola virus have been initiated.

Several studies have demonstrated to ability of mRNA vaccines to elicit protective immune responses against **influenza**. Petsch et al. were the first to demonstrate that ID administration of protamine-complexed non-replicating sequence-optimized mRNA vaccines encoding influenza HA was protective in mice upon homologous challenge with influenza H1N1, H3N2, and H5N1 and was immunogenic in ferrets and pigs (124). Furthermore, 10 μg of a comparable HA encoding vaccine delivered IM as LNP formulation elicited functional antibody responses in NHPs, that remained stable over a duration of one year, with HI titer remaining above 1:40 as the surrogate measure of protection in humans (141). A recently published study evaluated the efficacy of LNP-formulated, mRNA vaccines featuring modified nucleotides, that encoded for HA proteins of the potentially pandemic influenza A subtypes H10N8 or H7N9 (159). A single low dose (0.4–10 μg) of H7N9 mRNA vaccine applied ID or IM protected mice from a lethal homologous challenge and reduced lung viral titers were observed upon single-dose ID immunization of ferrets using 10–200 μg. In NHPs, both H10 and H7 mRNA vaccines tested at doses ranging from 200 to 400 μg generated robust HI titers after a single IM or ID immunization which were boosted following the second vaccination. However, upon both H10 and H7 immunization, NHPs that received the 400 μg dose experienced some systemic symptoms (e.g., warm to touch pain at the injection site, injection site irritation, and, in some cases, decreased food consumption) which resolved within 2–3 days. Interim results from a phase I first-in-human, randomized, double-blind, placebo-controlled, dose-ranging study of the H10N8 mRNA vaccine administered IM at a dose of 100 μg in healthy adult subjects (NCT03076385) showed high seroconversion rates, demonstrating robust prophylactic immunity in humans (Table 3). Adverse events were mild or moderate with only few severe and non-serious events. Of note, further clinical studies testing the efficacy of a comparable mRNA vaccine format against H7N9 (NCT03345043) and Chikungunya (NCT03325075) are currently ongoing with an estimated primary completion date in September 2018 and 2019, respectively. However, no details of these studies are available as yet.

Overall, these data show that non-replicating LNP-encapsulated mRNA vaccines can induce functional antibody titers at levels associated with protection with acceptable tolerability profiles upon parenteral administration. Future studies that employ LNPs for encapsulation of non-replicating mRNA targeting diverse and more complex antigens are required to demonstrate the broad applicability of this vaccine platform against pathogens posing potential pandemic threats.

## Conclusions

Pandemics such as HIV, Ebola, and Zika have raised the awareness of global threats to human health posed by known as well as newly emerging pathogens and can provide the impetus to prepare against future pandemics by promoting the development of vaccine platforms that can tackle the challenges of outbreak situations. New platforms, such as viral vector and nucleic acid based vaccines meet the prerequisites to provide solutions for some of these challenges by representing highly versatile technologies that allow fast vaccine manufacturing. Each vaccine technology has its own advantages and disadvantages related to its ability to induce certain immune responses, manufacturing capacity and safety for human use (Table 4). Viral vector based vaccines are able to induce potent immune responses against the encoded target antigen. Indeed, a number of clinical trials have demonstrated that viral vector based vaccines such as VSV-ZEBOV show great promise for inducing protective responses in humans. However, antigen delivery in the context of an unrelated virus renders this technology relatively complex in terms of manufacturing. Furthermore, the presence of immune targets other than the target antigen can lead to unfavorable effects such as pre-existing immunity hampering immune responses, as seen for Ad5 vectors, or the inability to use the same technology for repeated vaccinations. In addition, delivery of attenuated viral vectors raises safety concerns due to the risk of adverse events and residual viral replication upon delivery, as detected in a small number of subjects in a clinical trial testing VSV-ZEBOV. DNA based vaccines offer the advantage of allowing a relatively simple, fully synthetic production process. While the presence of non-functional sequences in original DNA vectors raised regulatory safety concerns, newer developments allow minimal constructs that exclusively encode for the target antigen. Several studies have demonstrated the safety of DNA vaccines for human use and clinical trials testing vaccines against influenza and Zika have furthermore highlighted the speed of vaccine development supported by this technology. However, the potential for long term persistence and genomic integration and the dependence on injection devices or electroporation represent some important disadvantages of this technology. Some, especially early, clinical studies testing DNA based vaccines have yielded somewhat discouraging results in terms of immunogenicity, while newer trials, such as studies testing DNA vaccines against Zika virus, have demonstrated that this technology is able to induce promising immune responses. Like DNA vaccines, RNA based vaccine technologies support a comparably simple, fully synthetic manufacturing process that allows production of different vaccines using the same established production process and facility. Their inability for genomic integration and lack of persistence in the cells of the vaccinee offers important advantages in terms of vaccine safety. However, since RNA vaccines represent the most recently developed technology described here, their use in humans is less well characterized than for viral vector or DNA based vaccines. Although further studies will be required to fully characterize this technology in humans, clinical studies conducted so far have yielded overall encouraging results in terms of safety and immunogenicity and provide support for further clinical exploration.

**Table 4:** Summarized properties of discussed vaccine technologies.

	**Viral vector based vaccines**	**DNA vaccines**	**RNA vaccines**
Platform versatility	+	+	+
Induction of cellular and humoral immune responses	+	+	+
Fully synthetic vaccine production possible	–	+	+
Delivery as minimal vaccine construct possible^*^	–	±	+
Repeated vaccine applications possible	±	+	+
Vaccine safety	±	+	++
Immunogenicity demonstrated in clinical studies	+	±	±

* *Minimal construct: the vaccine exclusively encodes the target antigen*.

While it seems unlikely that a single technology will be able to provide a solution for each future outbreak situation, the combination of present knowledge, ongoing development and the growing understanding of human immunology can provide tools to successfully combat emerging global threats.

## Author contributions

SR responsible for writing and coordination of the manuscript. EJ wrote part of the manuscript. KS wrote part of the manuscript. BP discussed the manuscript and reviewed the document.

### Conflict of interest statement

SR, EJ, KS, and BP are employed by CureVac AG.

## Footnotes

^1^http://www.iata.org/pressroom/pr/Pages/2017-02-02-01.aspx.

^2^http://www.who.int/en/news-room/fact-sheets/detail/hiv-aids.

^3^http://www.who.int/emergencies/mers-cov/en/.

^4^https://www.cdc.gov/vhf/ebola/outbreaks/history/chronology.html.

^5^http://www.who.int/csr/disease/ebola/en/.

^6^http://www.who.int/immunization/diseases/dengue/revised_SAGE_recommendations_dengue_vaccines_apr2018/en//.

^7^https://www.fda.gov/downloads/Guidances/UCM439273.pdf.

^8^https://www.fda.gov/downloads/biologicsbloodvaccines/guidancecomplianceregulatoryinformation/guidances/vaccines/ucm091968.pdf.

^9^http://www.who.int/biologicals/publications/trs/areas/vaccines/dna/Annex%201_DNA%20vaccines.pdf?ua=1.

^10^http://www.who.int/influenza/human_animal_interface/2017_07_25_tableH5N1.pdf.
